# Estimated Protection of Prior SARS-CoV-2 Infection Against Reinfection With the Omicron Variant Among Messenger RNA–Vaccinated and Nonvaccinated Individuals in Quebec, Canada

**DOI:** 10.1001/jamanetworkopen.2022.36670

**Published:** 2022-10-14

**Authors:** Sara Carazo, Danuta M. Skowronski, Marc Brisson, Chantal Sauvageau, Nicholas Brousseau, Rodica Gilca, Manale Ouakki, Sapha Barkati, Judith Fafard, Denis Talbot, Vladimir Gilca, Geneviève Deceuninck, Christophe Garenc, Alex Carignan, Philippe De Wals, Gaston De Serres

**Affiliations:** 1 Biological Risks Unit, Institut National de Santé Publique du Québec, Quebec City, Quebec, Canada; 2 Communicable Diseases and Immunization Services, British Columbia Centre for Disease Control, Vancouver, British Columbia, Canada; 3 Centre Hospitalier Universitaire de Québec-Université Laval Research Center, Quebec City, Quebec, Canada; 4 Social and Preventive Medicine Department, Faculty of Medicine, Laval University, Quebec City, Quebec, Canada; 5 Division of Infectious Diseases, Department of Medicine, McGill University Health Center, McGill University, Montreal, Quebec, Canada; 6 Laboratoire de Santé Publique du Québec, Institut National de Santé Publique du Québec, Sainte-Anne-de-Bellevue, Quebec, Canada; 7 Department of Microbiology and Infectious Diseases, Sherbrook University, Sherbrook, Quebec, Canada

## Abstract

**Importance:**

The Omicron variant is phylogenetically and antigenically distinct from earlier SARS-CoV-2 variants and the original vaccine strain. Protection conferred by prior SARS-CoV-2 infection against Omicron reinfection, with and without vaccination, requires quantification.

**Objective:**

To estimate the protection against Omicron reinfection and hospitalization conferred by prior heterologous non-Omicron SARS-CoV-2 infection and/or up to 3 doses of an ancestral, Wuhan-like messenger RNA (mRNA) vaccine.

**Design, Setting, and Participants:**

This test-negative, population-based case-control study was conducted between December 26, 2021, and March 12, 2022, and included community-dwelling individuals aged 12 years or older who were tested for SARS-CoV-2 infection in the province of Quebec, Canada.

**Exposures:**

Prior laboratory-confirmed SARS-CoV-2 infection with or without mRNA vaccination.

**Main Outcomes and Measures:**

The main outcome was laboratory-confirmed SARS-CoV-2 reinfection and associated hospitalization, presumed to be associated with the Omicron variant according to genomic surveillance. The odds of prior infection with or without vaccination were compared for case participants with Omicron infection and associated hospitalizations vs test-negative control participants. Estimated protection was derived as 1 − the odds ratio, adjusted for age, sex, testing indication, and epidemiologic week. Analyses were stratified by severity and time since last non-Omicron infection or vaccine dose.

**Results:**

This study included 696 439 individuals (224 007 case participants and 472 432 control participants); 62.2% and 63.9% were female and 87.4% and 75.5% were aged 18 to 69 years, respectively. Prior non-Omicron SARS-CoV-2 infection was detected for 9505 case participants (4.2%) and 29 712 control participants (6.3%). Among nonvaccinated individuals, prior non-Omicron infection was associated with a 44% reduction (95% CI, 38%-48%) in Omicron reinfection risk, which decreased from 66% (95% CI, 57%-73%) at 3 to 5 months to 35% (95% CI, 21%-47%) at 9 to 11 months postinfection and was below 30% thereafter. The more severe the prior infection, the greater the risk reduction. Estimated protection (95% CI) against Omicron infection was consistently significantly higher among vaccinated individuals with prior infection compared with vaccinated infection-naive individuals, with 65% (63%-67%) vs 20% (16%-24%) for 1 dose, 68% (67%-70%) vs 42% (41%-44%) for 2 doses, and 83% (81%-84%) vs 73% (72%-73%) for 3 doses. For individuals with prior infection, estimated protection (95% CI) against Omicron-associated hospitalization was 81% (66%-89%) and increased to 86% (77%-99%) with 1, 94% (91%-96%) with 2, and 97% (94%-99%) with 3 mRNA vaccine doses, without signs of waning.

**Conclusions and Relevance:**

The findings of this study suggest that vaccination with 2 or 3 mRNA vaccine doses among individuals with prior heterologous SARS-CoV-2 infection provided the greatest protection against Omicron-associated hospitalization. In the context of program goals to prevent severe outcomes and preserve health care system capacity, a third mRNA vaccine dose may add limited protection in twice-vaccinated individuals with prior SARS-CoV-2 infection.

## Key Points

**Question:**

How much does prior heterologous non-Omicron SARS-CoV-2 infection, with and without messenger RNA (mRNA) vaccination, reduce Omicron reinfection risk?

**Findings:**

In this test-negative case-control study with 696 439 participants aged 12 years and older, prior non-Omicron SARS-CoV-2 infection was associated with 44% and 81% reductions in the risk of Omicron infection and associated hospitalization, respectively. Protection against Omicron-associated hospitalization was sustained and improved among individuals who received 1 (86%), 2 (94%), or 3 (97%) mRNA vaccine doses.

**Meaning:**

These findings suggest that for twice-vaccinated individuals with prior non-Omicron SARS-CoV-2 infection, a third mRNA vaccine dose may add limited protection against Omicron-associated hospitalization.

## Teaser

This case-control study estimates protection conferred by prior SARS-CoV-2 infection against reinfection with the Omicron variant among community-dwelling messenger RNA–vaccinated and nonvaccinated individuals aged 12 years or older in Quebec, Canada.

## Introduction

In November 2021, the Omicron variant of SARS-CoV-2 emerged as a phylogenetically and antigenically distinct variant of concern (VOC). With its greater intrinsic transmissibility and capacity for immunologic escape, the Omicron variant has had unprecedented spread even among countries with high vaccine coverage, resulting in the most intense surge in infection since the beginning of the COVID-19 pandemic.^1,2,3,4,5,6^

Better understanding of the extent to which prior SARS-CoV-2 infection and/or vaccination may have modulated risk during the Omicron variant surge is needed. In this population-based analysis, we quantified estimated protection associated with prior heterologous SARS-CoV-2 infection against Omicron reinfection and hospitalization, stratified by the timing and severity of prior infection. We further assessed the protection of up to 3 doses of heterologous messenger RNA (mRNA) ancestral Wuhan-like vaccine against Omicron infection among individuals with or without prior SARS-CoV-2 infection.

## Methods

This test-negative case-control study was conducted under the legal mandate of the National Director of Public Health of Quebec under the Public Health Act, and thus participant consent was waived. The Research Ethics Board of Centre Hospitalier Universitaire de Québec-Université Laval Research Center also approved this study. The study followed the Strengthening the Reporting of Observational Studies in Epidemiology (STROBE) reporting guideline.

### Study Design

This study included community-dwelling residents aged 12 years or older in the province of Quebec, Canada. Specimens were collected between December 26, 2021, and March 12, 2022 (epidemiologic weeks 52 and 10, respectively), and SARS-CoV-2 infection was confirmed with nucleic acid amplification testing (NAAT).

Case participants had a positive SARS-CoV-2 test result during the study period. Individuals were censored upon first being identified as a COVID-19 case. Control participants had a negative SARS-CoV-2 test result during the study period. For individuals with multiple negative test results, a single negative specimen per individual was randomly selected.

### Data Sources

In Quebec, NAAT was limited to prioritized groups during the first wave of the COVID-19 pandemic but was broadly accessible to the general population through community-based assessment centers from July 1, 2020, to January 4, 2022. During the Omicron wave from January 5, 2022 onward, NAAT was restricted as a result of limited laboratory capacity and increased availability of rapid antigen detection testing (RADT). Nucleic acid amplification testing was available to participants consulting or admitted to the hospital, to health care workers and their families, and to residents in closed settings, but it was not routinely available to asymptomatic contacts or individuals with a mild case.^7^

Specimens were sampled from the provincial laboratory database, which includes all NAAT results for SARS-CoV-2 testing conducted in Quebec since the beginning of the pandemic. This database also captures testing indications, including the following categories: (1) symptomatic, tested in assessment centers; (2) symptomatic, in the emergency department or hospital; (3) symptomatic, among health care workers; (4) asymptomatic, during outbreaks in care facilities or closed settings; (5) asymptomatic, screening at hospital preadmission; (6) asymptomatic contacts; (7) other asymptomatic; and (8) any other reason, combined.

Specimens within the provincial laboratory database were linked to 4 data sources through unique personal identification numbers. These sources were as follows: (1) a provincial immunization registry that specifies vaccine status for all Quebec residents, including type, dates, and number of doses; (2) a database of all COVID-19 cases reported to the public health department, including demographic and clinical details; (3) an administrative hospitalization database; and (4) VOC screening and/or whole-genome sequencing results.

### Outcome and Primary Infection Definitions

The main outcome was any NAAT-confirmed SARS-CoV-2 infection during the study period. COVID-19 hospitalization was also assessed and was defined as admission for at least 24 hours and within 14 days after a positive SARS-CoV-2 test result. Participants with a positive test result after hospitalization or during screening at hospital preadmission (according to testing indication) were excluded.

Reinfection was defined as the first positive SARS-CoV-2 result during the study period identified 90 days or more after a primary infection (PI).^8^ Primary infection was defined as the first positive specimen from the beginning of the pandemic. Individuals without such records in the laboratory database were considered to not have a prior infection (or infection naive). Clinical information from the COVID-19 database was used to ascertain symptom presence and PI severity (asymptomatic, symptomatic, or hospitalization). We excluded individuals for whom SARS-CoV-2 reinfection was documented before December 26, 2021, and all specimens collected within 90 days after a first positive specimen.

### Variant of Concern Attribution

Variant of concern detection varied provincially in response to changing epidemic patterns, case load, and laboratory capacity and to the profile of emerging VOCs (eFigure 1 in the Supplement).^9^ For test-positive specimens collected between February 1 and October 8, 2021, Alpha, Beta, Gamma, or Delta VOC status was based on individual-level genetic characterization undertaken on all SARS-CoV-2 detections. Variant-of-concern status was otherwise presumptive based on provincial-level genomic surveillance. Primary infections were designated as pre-VOC (occurring before February 1, 2021) or Delta (October 9 to December 12, 2021, the last day of specimen collection meeting the ≥90-day reinfection definition). All infections during the study period from December 26, 2021, to March 12, 2022, were assumed to be attributable to the Omicron variant.

### Vaccination Definitions

The publicly funded COVID-19 immunization campaign in Quebec began on December 14, 2020, with second vaccine doses deferred up to 16 weeks and third doses beginning in December 2021 (eFigure 1 in the Supplement). Community-based vaccination sequentially prioritized the oldest individuals to the youngest down to age 5 years.^10,11^

Vaccination was defined at the specimen collection date. Single-dose mRNA vaccination was defined as receipt of 1 dose of the BNT162b2 (Pfizer-BioNTech) or mRNA-1273 (Moderna) vaccine at least 21 days before specimen collection to ensure a full primary immune response. Vaccination with 2 or 3 doses was defined as receipt at least 7 days before specimen collection.^12,13^ To exclude immunocompromised persons for whom a 4-week interval between the second and third doses was recommended,^14^ only participants receiving the third dose 90 days or more after the second dose were included per the more routine recommendation in Quebec.^15^

### Exclusion Criteria

Specimens were excluded if they met any of the following criteria: if the reason for testing was to confirm COVID-19 recovery or a positive RADT result, if the vaccination date or interval between doses was invalid, if the first dose was received earlier than 21 days before testing or the second or third doses were received earlier than 7 days before testing, or if a non-mRNA vaccine such as AZD1222 (AstraZeneca) or Ad26.COV2.S (Johnson & Johnson/Janssen) was received.

### Statistical Analysis

Data were analyzed from March 18, 2022, to April 15, 2022. Exposure was analyzed as a combination of prior PI and vaccine receipt. Categories were as follows: PI, nonvaccinated (PI-NV); PI before the first, second, or third vaccine dose (PI-V1, PI-V2, or PI-V3); PI after the first, second, or third vaccine dose (V1-PI, V2-PI, or V3-PI); PI after the first but before the second vaccine dose (V1-PI-V2); PI after the first but before the second and third vaccine doses (V1-PI-V3); and PI after the second but before the third vaccine dose (V2-PI-V3). Individuals with no prior infection were also subcategorized as NI-NV, NI-V1, NI-V2, and NI-V3 based on the number of vaccine doses received (eFigure 2 in the Supplement).

The main comparator was the NI-NV group. The odds of having experienced PI with or without vaccination (PI-NV, PI-V1, PI-V2, or PI-V3) or of NI with vaccination (NI-V1, NI-V2, or NI-V3) were compared among case participants with Omicron infection or associated hospitalization vs test-negative control participants. Post hoc analyses compared vaccinated individuals with prior infection (PI-V1, PI-V2, or PI-V3) with the PI-NV group. Adjusted odds ratios (ORs) were derived from logistic regression models controlling for age, sex, testing indication, and epidemiologic week. The same set of control participants was used to assess protection against infection and hospitalization. Estimated protection was derived as 1 − adjusted OR with 95% CIs.

Analyses were stratified by age group, PI severity and variant status, time since PI, and time since last event (PI or vaccine dose). Participants with prior infection (PI-NV, PI-V1, PI-V2, or PI-V3) were also directly compared with vaccinated individuals with no prior infection (NI-V1, NI-V2, or NI-V3). Statistical analyses were performed using SAS, version 9.4 (SAS Institute Inc).

## Results

### Study Population

Among 1 778 623 NAAT specimens obtained during the study period, data linkage was successful for 1 754 358 (98.6%). After eligibility criteria and exclusions were applied (eFigure 3 in the Supplement) and 1 negative specimen per individual was randomly selected as the control, there were 696 439 individuals (224 007 test-positive case participants and 472 432 test-negative control participants) included in the analyses.

### Baseline Characteristics

Table 1 presents the baseline characteristics of case and control participants by prior PI status. Of the 224 007 case participants and 472 432 control participants, most were female (62.2% vs 63.9%) and were aged 18 to 69 years (87.4% vs 75.5%).

**Table 1.** Characteristics of Omicron Case and Control Participants Stratified by Primary SARS-CoV-2 (Non–Omicron Variant) Infection History

Characteristic	Participant group			
	Cases (n = 224 007), No. (%)		Controls (n = 472 432), No. (%)	
	Prior non-Omicron PI (n = 9505)	No prior infection (n = 214 502)	Prior non-Omicron PI (n = 29 712)	No prior infection (n = 442 720)
Sex				
Female	6681 (70.3)	132 726 (61.9)	20 668 (69.6)	281 290 (63.5)
Male	2824 (29.7)	81 776 (38.1)	9044 (30.4)	161 430 (36.5)
Age, y				
12-17	321 (3.4)	11 940 (5.6)	886 (3.0)	13 756 (3.1)
18-49	7120 (74.9)	137 679 (64.2)	18 845 (63.4)	211 324 (47.7)
50-69	1789 (18.8)	49 302 (23.0)	7668 (25.8)	119 015 (26.9)
≥70	275 (2.9)	15 581 (7.3)	2313 (7.8)	98 625 (22.3)
Health care worker	3821 (40.2)	45 251 (21.1)	12 952 (43.6)	98 571 (22.3)
Year and epidemiologic week of specimen collection (calendar start and end dates)				
2021-2022				
52 (Dec 26-Jan 1)	2599 (27.3)	70 753 (33.0)	6109 (20.6)	90 962 (20.6)
2022				
1 (Jan 2-8)	2460 (25.9)	54 928 (25.6)	5255 (17.7)	71 372 (16.1)
2-4 (Jan 9-29)	2583 (27.2)	46 624 (21.7)	8735 (29.4)	117 801 (26.6)
5-7 (Jan 30-Feb 19)	1258 (13.2)	27 980 (13.0)	5763 (19.4)	91 917 (20.8)
8-10 (Feb 20-Mar 12)	605 (6.4)	14 217 (6.6)	3850 (13.0)	70 668 (16.0)
Testing indication				
Symptomatic				
Assessment center	3233 (34.0)	102 260 (47.7)	4050 (13.6)	74 839 (16.9)
Emergency department	284 (3.0)	12 551 (5.9)	2000 (6.7)	46 267 (10.5)
Health care worker	3262 (34.3)	49 111 (22.9)	4117 (13.9)	46 454 (10.5)
Asymptomatic				
Closed-setting outbreak	1009 (10.6)	13 935 (6.5)	7776 (26.2)	88 078 (19.9)
Hospital preadmission	270 (2.8)	8157 (3.8)	4450 (15.0)	102 848 (23.2)
Contact	687 (7.2)	17 095 (8.0)	2608 (8.8)	39 056 (8.8)
Other	505 (5.3)	5406 (2.5)	3396 (11.4)	28 075 (6.3)
Other reasons combined	255 (2.7)	5987 (2.8)	1315 (4.4)	17 103 (3.9)
Prior PI VOC status				
Pre-VOC	7424 (78.1)	NA	22 826 (76.8)	NA
Alpha	613 (6.5)	NA	1955 (6.6)	NA
Delta	253 (2.7)	NA	1458 (4.9)	NA
Other and/or unknown^a^	1215 (12.8)	NA	3427 (11.7)	NA
Interval between PI and specimen collection, d (mo)				
90-182 (3-5)	372 (3.9)	NA	1791 (6.0)	NA
183-274 (6-8)	590 (6.2)	NA	1687 (5.7)	NA
275-364 (9-11)	1904 (20.0)	NA	4931 (16.6)	NA
365-547 (12-18)	4747 (49.9)	NA	14 355 (48.3)	NA
548-730 (19-24)	1892 (19.9)	NA	6948 (23.4)	NA
Median (IQR), d	407 (354-480)	NA	414 (356-514)	NA
Range, d	90-714	NA	90-731	NA
Prior PI severity				
Asymptomatic	1183 (12.5)	NA	2685 (9.0)	NA
Symptomatic				
Nonhospitalized	8123 (85.5)	NA	25 615 (86.2)	NA
Hospitalized	199 (2.1)	NA	1412 (4.8)	NA

Abbreviations: NA, not applicable; PI, primary infection; VOC, variant of concern.

^a^ Other and/or unknown indicates PI during periods with mixed VOC circulation without individual-level genotyping or identified to be Beta (n = 2) or Gamma (n = 5).

Among case participants identified during the study period, there were 9505 (4.2%) reinfections (Table 2). With respect to case participant vaccination status, 17 633 (7.9%) overall were NI-NV but most (142 326 [63.5%]) were NI-V2. Conversely, 915 (0.4%) were PI-NV and 347 (0.2%) and 8243 (3.7%) were vaccinated before and after PI, respectively. Among the 5057 case participants with COVID-19 hospitalizations during the study period, there were 64 (1.3%) with prior PIs; no deaths were identified among case participants with prior PI.

**Table 2.** Omicron Variant Case and Control Participants Stratified by Outcome Severity and Primary SARS-CoV-2 (Non–Omicron Variant) Infection and Vaccination History

Exposure	Participant group				
	Cases (n = 224 007), No. (%)			Controls (n = 472 432), No. (%)	All (n = 696 439), No. (%)
	Infections (n = 224 007)	Hospitalizations (n = 5057)^a^	Deaths (n = 920)^a^		
Prior (non-Omicron) SARS-CoV-2 PI	9505 (4.2)	64 (1.3)	0	29 712 (6.3)	39 217 (5.6)
PI-NV	915 (0.4)	13 (0.3)	0	1817 (0.4)	2732 (0.4)
Vaccination after PI	8243 (3.7)	48 (0.9)	0	26 006 (5.5)	34 249 (4.9)
PI-V1	2102 (0.9)	18 (0.4)	0	4906 (1.0)	7008 (1.0)
PI-V2	5038 (2.3)	23 (0.5)	0	13 942 (3.0)	18 980 (2.7)
PI-V3	1103 (0.5)	7 (0.1)	0	7158 (0.2)	8261 (1.2)
Vaccination before PI	347 (0.2)	3 (0.1)	0	1889 (0.4)	2236 (0.3)
V1-PI	13 (<0.1)	0	0	54 (<0.1)	67 (<0.1)
V2-PI	107 (0.1)	1 (<0.1)	0	474 (0.1)	581 (0.1)
V3-PI	2 (<0.1)	0	0	5 (<0.1)	7 (<0.1)
V1-PI-V2	123 (0.1)	1 (<0.1)	0	385 (0.1)	508 (0.1)
V1-PI-V3	66 (<0.1)	1 (<0.1)	0	485 (0.1)	551 (0.1)
V2-PI-V3	36 (<0.1)	0	0	486 (0.1)	522 (0.1)
NI with prior SARS-CoV-2 infection					
Any	214 502 (95.8)	4993 (98.7)	920 (100)	442 720 (93.7)	657 222 (94.4)
NI-NV	17 633 (7.9)	1399 (27.7)	240 (26.1)	20 997 (4.4)	38 630 (5.6)
NI-V1	3899 (1.7)	146 (2.9)	32 (3.5)	5307 (1.1)	9206 (1.3)
NI-V2	142 326 (63.5)	2140 (42.3)	377 (41.0)	185 780 (39.3)	328 106 (47.1)
NI-V3	50 644 (22.6)	1308 (25.9)	271 (29.5)	230 636 (48.8)	281 280 (40.4)

Abbreviations: NI, no prior infection; NI-NV, no prior infection, nonvaccinated; NI-V1, NI-V2, or NI-V3, no prior infection, 1, 2, or 3 vaccine doses; PI, primary infection; PI-NV, prior infection, nonvaccinated; PI-V1, prior infection before 1 vaccine dose; PI-V2, prior infection before 2 vaccine doses; PI-V3, prior infection before 3 vaccine doses; V1-PI, prior infection after 1 vaccine dose; V1-PI-V2, prior infection after the first but before the second vaccine dose; V1-PI-V3, prior infection after the first but before the second and third vaccine doses; V2-PI, prior infection after 2 vaccine doses; V2-PI-V3, prior infection after the second but before third vaccine dose; V3-PI, prior infection after 3 vaccine doses.

^a^ Hospitalizations (n = 1749) and deaths (n = 139) among participants who had “preadmission to a health facility” as a testing indication were excluded.

Primary infection was identified in 29 712 of 472 432 control participants (6.3%) during the study period (Table 2). With respect to control participant vaccination status, 20 997 (4.4%) were NI-NV but most (230 636 [48.8%]) were NI-V3. Conversely, 1817 (0.4%) were PI-NV and 1889 (0.4%) and 26 006 (5.5%) were vaccinated before and after PI, respectively.

Of the 9505 case participants with reinfection, 7424 (78.1%) prior PIs were genetically categorized as pre-VOC. This result likely reflects a longer period for accrual (notably, prevaccine rollout) compared with Alpha, Delta, or other or unknown VOC circulation, which comprised 613 (6.5%), 253 (2.7%), and 1215 (12.8%) PIs, respectively (Table 1). The median (IQR) specimen collection interval between PI and case or control detection was similar (407 [354-480] vs 414 [356-514] days, respectively).

### Prior Infection–Associated Protection Against Omicron Reinfection, Without Vaccination

Without vaccination, non-Omicron PI was associated with a 44% reduction (95% CI, 38%-48%) in Omicron reinfection risk (Table 3). The more severe the prior infection, the greater the Omicron risk reduction. Estimated protection (95% CI) of 8% (−17%-28%), 43% (37%-49%), and 68% (51%-80%) for prior asymptomatic, symptomatic ambulatory, or hospitalized infections was also evident among vaccinated individuals. Protection associated with asymptomatic infection alone was evident for the first 6 months (49% [95% CI, 8%-72%]) but not thereafter (Table 4).

**Table 3.** Primary SARS-CoV-2 (Non–Omicron Variant) Infection–Induced Estimated Protection Against Omicron Reinfection and Hospitalization Among Vaccinated Individuals (by Number of Doses), Relative to Nonvaccinated Individuals With or Without Infection History

Exposure	Estimated protection, % (95% CI)^a^			
	Omicron infection		Omicron-associated hospitalization	
	Unadjusted	Adjusted	Unadjusted	Adjusted
**Compared with nonvaccinated individuals without prior infection**				
No prior infection				
Unvaccinated	1 [Reference]	1 [Reference]	1 [Reference]	1 [Reference]
Vaccinated				
1 dose	12 (8 to 16)	20 (16 to 24)	60 (53 to 67)	52 (42 to 61)
2 doses	9 (7 to 11)	42 (41 to 44)	86 (85 to 87)	76 (74 to 78)
3 doses	74 (73 to 74)	73 (72 to 73)	92 (92 to 93)	91 (91 to 92)
Prior primary infection				
Unvaccinated	40 (35 to 45)	44 (38 to 48)	90 (83 to 94)	81 (66 to 89)
Vaccinated				
1 dose	49 (46 to 52)	65 (63 to 67)	96 (93 to 97)	86 (77 to 91)
2 doses	57 (55 to 59)	68 (67 to 70)	98 (97 to 99)	94 (91 to 96)
3 doses	82 (80 to 83)	83 (81 to 84)	99 (98 to 99)	97 (94 to 99)
**Compared with nonvaccinated individuals with prior infection**				
Prior primary infection				
Unvaccinated	1 [Reference]	1 [Reference]	1 [Reference]	1 [Reference]
Vaccinated				
1 dose	15 (6 to 23)	40 (33 to 46)	57 (13 to 79)	25 (−56 to 64)
2 doses	28 (22 to 34)	45 (40 to 50)	81 (63 to 91)	70 (39 to 85)
3 doses	69 (66 to 72)	70 (67 to 73)	89 (71 to 95)	85 (62 to 94)

^a^ Logistic regression models were adjusted for age (12-17, 18-49, 50-69, and ≥70 years), sex, testing indication, and epidemiologic week.

**Table 4.** Primary Non-Omicron SARS-CoV-2 Infection–Induced Estimated Protection Against Omicron Reinfection Among Vaccinated and Nonvaccinated Individuals (by Number of Doses After Primary Infection) Stratified by Age and Primary Infection Characteristics, Relative to Nonvaccinated Individuals With No Infection History

Characteristic	Adjusted estimated protection, % (95% CI)^a^			
	PI-NV	PI-V1	PI-V2	PI-V3
Total	44 (38 to 48)	65 (63 to 67)	68 (67 to 70)	83 (81 to 84)
Age, y				
12-17	57 (36 to 71)	78 (70 to 83)	79 (74 to 93)	96 (65 to 99)
18-49	44 (29 to 43)	62 (60 to 65)	67 (65 to 68)	79 (77 to 81)
50-69	51 (38 to 60)	71 (66 to 75)	72 (69 to 74)	86 (83 to 88)
≥70	46 (16 to 65)	79 (65 to 87)	67 (60 to 73)	81 (75 to 86)
VOC status of prior PI				
Pre-VOC	29 (20 to 37)	62 (59 to 64)	67 (65 to 69)	81 (79 to 82)
Alpha	44 (26 to 57)	73 (68 to 78)	75 (71 to 79)	92 (84 to 96)
Delta	67 (57 to 75)	73 (61 to 82)	87 (70 to 94)	NE
Other and/or unknown^b^	50 (38 to 60)	70 (64 to 74)	69 (65 to 72)	86 (81 to 90)
Prior PI severity				
Asymptomatic	8 (−17 to 28)	40 (28 to 50)	43 (36 to 49)	66 (59 to 73)
Symptomatic				
Nonhospitalized	43 (37 to 49)	66 (64 to 68)	70 (68 to 71)	82 (81 to 84)
Hospitalized	68 (51 to 80)	79 (67 to 86)	77 (72 to 82)	87 (80 to 91)
Interval since prior PI, mo				
3-5	66 (57 to 73)	73 (64 to 80)	88 (76 to 94)	NE
6-8	49 (32 to 61)	76 (70 to 80)	78 (74 to 81)	89 (70 to 96)
9-11	35 (21 to 47)	65 (61 to 69)	68 (65 to 70)	85 (81 to 89)
12-18	29 (17 to 38)	61 (58 to 64)	67 (65 to 69)	80 (78 to 82)
19-24	27 (8 to 42)	61 (55 to 67)	67 (64 to 70)	80 (78 to 82)
Severity of and interval since prior PI, mo				
Asymptomatic				
<6	49 (8 to 72)	66 (17 to 86)	NE	NE
≥6	−5 (−36 to 20)	39 (26 to 49)	42 (35 to 49)	66 (59 to 73)
Symptomatic				
Nonhospitalized				
<6	67 (57 to 74)	73 (63 to 81)	88 (76 to 94)	NE
≥6	37 (29 to 43)	66 (63 to 68)	69 (68 to 71)	82 (81 to 84)
Hospitalized				
<6	81 (52 to 92)	84 (23 to 97)	73 (−162 to 97)	NE
≥6	62 (36 to 77)	78 (66 to 86)	77 (72 to 82)	87 (80 to 91)
Interval since last vaccination, mo^c^				
<2	NA	81 (74 to 86)	82 (80 to 84)	83 (81 to 84)
2-5	NA	64 (60 to 67)	67 (65 to 68)	80 (76 to 84)
6-8	NA	62 (58 to 65)	63 (60 to 65)	NE
9-11	NA	61 (54 to 67)	62 (42 to 75)	NE
12-14	NA	65 (48 to 76)	NE	NE

Abbreviations: NA, nonapplicable; NE, nonestimable; PI, primary infection; PI-NV, prior infection nonvaccinated; PI-V1, prior infection before 1 vaccine dose; PI-V2, prior infection before 2 vaccine doses; PI-V3, prior infection before 3 vaccine doses; VOC, variant of concern.

^a^ Logistic regression models compared vaccinated and unvaccinated persons with prior PI with unvaccinated individuals without prior PI. All estimates were adjusted for age (12-17, 18-49, 50-69, and ≥70 years), sex, testing indication, and epidemiologic week.

^b^ Case participants without genotyping during periods with mixed circulation or case participants with the Beta (n = 2) or Gamma (n = 5) variant.

^c^ Models stratified for delay from last vaccination were not adjusted for epidemiologic week because of an insufficient number of case participants in each stratum and high correlation between delay and epidemiologic week for those vaccinated with 3 doses.

Protection associated with PI varied by VOC status. Estimated adjusted protection (95% CI) was 29% (20%-37%), 44% (26%-57%), and 67% (57%-75%) for pre-VOC, Alpha, and Delta, respectively (Table 4). However, this result may also reflect waning over differential time since variant-specific circulation. Estimated protection decreased from 66% (57%-73%) at 3 to 5 months postinfection, reflecting the more proximal Delta period, to 49% (32%-61%) at 6 to 8 months when Alpha foremost contributed, and to 35% (21%-47%) at 9 to 11 months and then remained below 30% thereafter, reflecting the more distant pre-VOC period (eFigure 4 in the Supplement).

### Estimated Vaccine Protection Against Omicron Reinfection

Estimated vaccine protection against Omicron infection was consistently significantly higher among vaccinated individuals with prior infection vs vaccinated but infection-naive individuals. Adjusted estimated protection (95% CI) was 65% (63%-67%) vs 20% (16%-24%) for 1 dose, 68% (67%-70%) vs 42% (41%-44%) for 2 doses, and 83% (81%-84%) vs 73% (72%-73%) for 3 doses (Table 3). For the same number of doses, protection against reinfection was similar whether the prior infection came before, between, or after vaccination (Figure).

**Figure.**
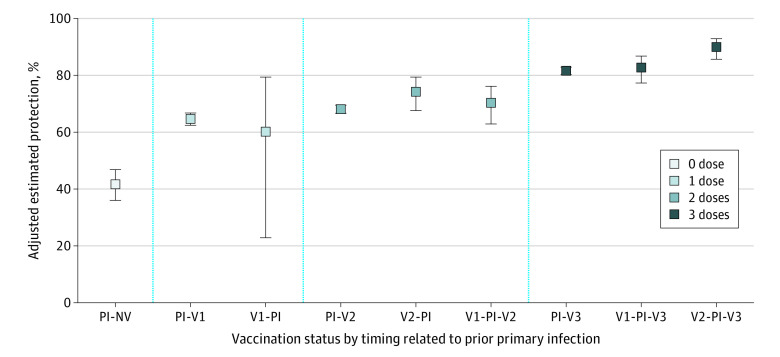
Prior SARS-CoV-2 Infection and Messenger RNA Vaccine Effectiveness Against Omicron Reinfection in Quebec, Canada, by Number of Doses and Timing Logistic regression models compared vaccinated and unvaccinated persons with prior infection (PI) with unvaccinated individuals without PI. All estimates were adjusted for age (12-17, 18-49, 50-69, and ≥70 years), sex, testing indication, and epidemiologic week. PI-NV indicates prior infection, nonvaccinated; PI-V1, PI-V2, or PI-V3, prior infection before 1, 2, or 3 vaccine doses, respectively; V1-PI, prior infection after 1 vaccine dose; V1-PI-V2, prior infection after the first but before the second vaccine dose; V1-PI-V3, prior infection after the first but before the second and third vaccine doses; V2-PI, prior infection after 2 vaccine doses; V2-PI-V3, prior infection after the second but before the third vaccine dose. Error bars indicate 95% CIs.

Receipt of 2 vaccine doses was associated with significantly lower adjusted estimated protection (95% CI) than 3 doses among both individuals with (68% [67%-70%] vs 83% [81%-84%]) and without (42% [41%-44%] vs 73% [72%-73%]) prior infection, recognizing longer median follow-up time (IQR) since the second vs third dose among both individuals with (158 [122-185] vs 27 [16-46] days) and without (173 [148-194] vs 37 [20-60] days) prior infection (not shown). Compared with unvaccinated individuals with prior infection, receipt of 1, 2, or 3 vaccine doses was associated with adjusted estimated protection (95% CI) of 40% (33%-46%), 45% (40%-50%), and 70% (67%-73%), respectively (Table 3).

Among individuals with prior infection, lower estimated protection after 1 or 2 vs 3 doses may be attributed in part to waning over differential time since vaccination. When standardized for the first 2 months postvaccination, adjusted estimated protection (95% CI) was similar for 1, 2, and 3 doses at 81% (74%-86%), 82% (80%-84%), and 83% (81%-84%), respectively, compared with unvaccinated infection-naive individuals and 67% (55%-76%), 72% (67%-76%), and 73% (69%-76%), respectively, compared with unvaccinated individuals with prior infection. At 2 to 5 months and compared with NI-NV individuals, 1- and 2-dose adjusted estimated protection (95% CI) was 64% (60%-67%) and 67% (65%-68%), which was lower than the 3-dose estimate (80% [76%-84%]), and ranged from 60% to 65% among 2-dose recipients thereafter. Similar differences were observed in estimated vaccine protection relative to PI-NV (Table 4; eFigure 4 and eTable in the Supplement).

### Infection and/or Vaccine-Associated Protection Against Omicron-Associated Hospitalization

Without vaccination, prior non-Omicron infection was associated with an 81% (95% CI, 66%-89%) reduction in Omicron-associated hospitalization risk (Table 3). Given the high level of infection-associated protection and vaccine coverage, the sample size was limited to further stratify by time since or severity of prior infection.

Estimated protection against Omicron-associated hospitalization was consistently significantly higher among vaccinated individuals with prior infection vs vaccinated infection-naive individuals. Adjusted estimated protection (95% CI) was 86% (77%-91%) vs 52% (42%-61%) for 1 dose, 94% (91%-96%) vs 76% (74%-78%) for 2 doses, and 97% (94%-99%) vs 91% (91%-92%) for 3 doses.

With regard to hospitalization, adjusted estimated protection (95% CI) for 2 doses was similar to 3 doses among individuals with prior infection compared with NI-NV (94% [91%-96%] vs 97% [94%-99%]) and was nonsignificantly lower compared with PI-NV (70% [39%-85%] vs 85% [62%-94%]). This result recognizes longer median follow-up time since the second vs third dose, as discussed earlier (Table 3).

Among individuals with prior infection, 2-dose estimated protection (95% CI) against hospitalization was similar at less than 6 months and at 6 to 11 months postvaccination (95% [92%-97%] vs 93% [86%-96%]). Among individuals without prior infection, a significant decline in 2-dose estimated vaccine protection was observed (81% [79%-83%] vs 73% [71%-75%], respectively).

## Discussion

In this case-control study, we observed that a single prior heterologous, non-Omicron SARS-CoV-2 infection, without vaccination, was associated with a reduction in subsequent Omicron reinfection risk by nearly half. In addition, this reduction was as much as two-thirds during the first 6 months and one-third 9 to 11 months after the prior PI. The more severe the PI, the greater the cross-protection against Omicron. Prior infection alone was associated with an approximately 80% reduction in Omicron-associated hospitalization risk, but better protection was associated with the combined presence of prior infection and vaccination (ie, hybrid protection), reaching 94% with 2 doses and improving only marginally to 97% with 3 doses. Overall, for the same number of vaccine doses, prior infection was associated with approximately 70% to 80% better protection against hospitalization compared with no prior infection. These observations suggest that absent prior infections and/or vaccination, the population impact of the Omicron surge may have been much worse.

Epidemiologic studies conducted during the pre-Omicron period showed that prior infection (without vaccination) reduced the risk of reinfection with the same or different variants by 80% to 100%, with minimal waning after 1 year.^16,17,18,19,20^ Few studies have quantified prior infection-induced protection against Omicron, but our findings seem consistent with those available. In Qatar, prior infection was reported to be 56% effective against Omicron reinfection and 88% effective against hospitalization, which was comparable against reinfection at 3 to 8 months (64%) and 15 months or more (60%) postinfection.^21^ A Czech study reported a decline in infection-induced protection against reinfection from 68% at 2 to 6 months to 13% more than 6 months later, although protection against hospitalization was 87% and well maintained.^22^ Overall, strong and sustained protection from prior heterologous infection against severe Omicron outcomes, including the absence of Omicron-associated deaths among survivors of prior infection, seems to be a common interpretation across the few available studies to date, including ours.^21,22^

One contribution of this study is our demonstration that prior asymptomatic infection was also protective, reducing Omicron reinfection risk by approximately half during the first 6 months. Thereafter, unvaccinated individuals with asymptomatic infection had no protective advantage compared with infection-naive individuals. During the pre-Omicron period, the findings of a Danish cohort study suggested that individuals with prior infection who experienced prior asymptomatic vs symptomatic infection had a 50% higher risk of Delta reinfection.^23^

Our findings suggest that prior heterologous SARS-CoV-2 infection conferred protection against Omicron reinfection (44%) and hospitalization (81%) that was comparable to outcomes after 2 doses of heterologous mRNA vaccine among individuals without prior infection (42% and 76%, respectively). Similar observations have been reported elsewhere during the pre-Omicron period, although these were not fully consistent across studies.^19,24,25,26,27,28,29^ During the Omicron period, 2 studies from Qatar suggested that higher protection against symptomatic Omicron reinfection was conferred by prior infection vs 2-dose vaccination.^30,31^ In contrast, investigators from the Netherlands reported lower protection from prior infection (25% [95% CI, 21%-29%]) vs 2-dose vaccination (33% [31%-35%]).^32^ Additional studies are needed to clarify these comparisons, recognizing the challenge in standardization based on time since last event (infection or vaccination).

In immunogenicity studies conducted during the pre-Omicron period, investigators reported that a single BNT162b2 vaccine dose among individuals with prior infection elicited robust antibody and T-cell responses, exceeding the 2-dose response in infection-naive individuals.^33,34,35,36^ In epidemiologic studies conducted before the emergence of the Omicron variant, hybrid immunity with 1 or 2 mRNA doses similarly reduced pre-Omicron reinfection risk by 60% to 80% compared with natural immunity, which persisted for up to 9 months,^18,37,38^ and exceeded 90% relative to unvaccinated infection-naive individuals.^16^

Perhaps not unexpectedly, we observed that prior infection improved vaccine protection (and vice versa) during the Omicron period. This result is consistent with the albeit limited epidemiologic evidence elsewhere pertaining to hybrid protection against Omicron infection.^22,36,39^ Among US health care workers with prior infection, 2-dose mRNA vaccination reduced the risk of symptomatic Omicron reinfection by 64% relative to the once or never-vaccinated (combined) group, similar to our 2-dose estimate of 68% relative to infection-naive, never-vaccinated individuals.^39^ Among US adults with prior infection, Omicron-associated hospitalization risk was reduced among the vaccinated vs unvaccinated by 33%, 35%, and 68% with 1, 2, and 3 mRNA doses, respectively.^40^ The latter contrasts with our findings suggesting that among individuals with prior infection, a third dose did not meaningfully improve the already-substantial 2-dose protection against Omicron-associated hospitalization (97% vs 94%) that was furthermore sustained for more than 1 year.

### Limitations

This study has some limitations. Notably, unrecognized or undocumented PIs may have led to underestimation of infection-induced protection, particularly for pre-VOC infections during the first wave when test access was limited. RADT, which was only available to the general population during the Omicron wave, may have reduced the detection of NAAT-confirmed infections, but this lack of sensitivity is unlikely to have biased the estimates.^41^ Although we could not control for the bias of differential virus exposure,^42^ similar patterns identified when protected groups were compared directly (eg, prior infection and vaccinated vs no infection and vaccinated) are reassuring. Immunocompromised individuals were prioritized for an early third dose in Quebec.^14^ To limit underestimation of 3-dose estimated vaccine protection associated with their potentially suboptimal immune responses,^43,44^ we excluded specimens from people revaccinated at an interval of less than 90 days between the second and third doses. Our results apply to individuals who survived PI; less than 1% of NAAT-confirmed case participants died, so their exclusion did not meaningfully influence our estimates. Our findings reflect heterologous infection- and/or vaccine-induced protection; homologous protection is anticipated to be higher.^45^ It was not possible to distinguish variation in protection based on VOC-specific PI vs time since PI because they were highly correlated.

## Conclusions

The findings of this case-control study suggest that vaccination with 2 or 3 doses among individuals with prior heterologous SARS-CoV-2 infection provided the greatest protection against severe outcomes as a result of Omicron reinfection. In the context of program goals to prevent severe outcomes and preserve health care system capacity, our findings further suggest that a third mRNA vaccine dose may add limited protection in twice-vaccinated individuals with prior infection. Pending vaccine updates, such doses may be better prioritized to more vulnerable individuals globally.

## Notes

Supplement. **eTable.** Estimated Vaccine Protection Against Omicron Reinfection Among Individuals With Prior SARS-CoV-2 Infection Stratified by Age and Primary Infection Characteristics, Relative to Nonvaccinated Individuals With Primary SARS-CoV-2 Infection **eFigure 1.** Epidemiologic Curve of Reported SARS-CoV-2 Cases and Vaccination Coverage in Quebec, Canada, Displayed by Month and Predominant Variant-of-Concern Periods **eFigure 2.** Exposure Categories Defined by Prior Primary SARS-CoV-2 Infection and Vaccination Histories **eFigure 3.** Participant Flowchart **eFigure 4.** Estimated Protection of Primary SARS-CoV-2 (Non-Omicron) Infection Against Omicron Reinfection, by Time Since Last Event (Primary Infection or Vaccination), Relative to No Vaccination and No Infection History
