# Supplementary material for: Estimated Protection of Prior SARS-CoV-2 Infection Against Reinfection With the Omicron Variant Among Messenger RNA–Vaccinated and Nonvaccinated Individuals in Quebec, Canada
Source: JAMA Netw Open. 2022 Oct 14;5(10):e2236670. doi: 10.1001/jamanetworkopen.2022.36670 (PMC9568797; doi:10.1001/jamanetworkopen.2022.36670)
Supplement: Supplement. — eTable. Estimated Vaccine Protection Against Omicron Reinfection Among Individuals With Prior SARS-CoV-2 Infection Stratified by Age and Primary Infection Characteristics, Relative to Nonvaccinated Individuals With Primary SARS-CoV-2 Infection eFigure 1. Epidemiologic Curve of Reported SARS-CoV-2 Cases and Vaccination Coverage in Quebec, Canada, Displayed by Month and Predominant Variant-of-Concern Periods eFigure 2. Exposure Categories Defined by Prior Primary SARS-CoV-2 Infection and Vaccination Histories eFigure 3. Participant Flowchart eFigure 4. Estimated Protection of Primary SARS-CoV-2 (Non-Omicron) Infection Against Omicron Reinfection, by Time Since Last Event (Primary Infection or Vaccination), Relative to No Vaccination and No Infection History [file jamanetwopen-e2236670-s001.pdf]

Supplementary Online Content

Carazo S, Skowronski DM, Brisson M, et al. Estimated protection of prior SARS-CoV-2 infection against reinfection with the Omicron variant among messenger RNA–vaccinated and nonvaccinated individuals in Quebec, Canada. *JAMA Netw Open*. 2022;5(10):e2236670. doi:10.1001/jamanetworkopen.2022.36670

eTable. Estimated Vaccine Protection Against Omicron Reinfection Among Individuals With Prior SARS-CoV-2 Infection Stratified by Age and Primary Infection Characteristics, Relative to Nonvaccinated Individuals With Primary SARS-CoV-2 Infection

eFigure 1. Epidemiologic Curve of Reported SARS-CoV-2 Cases and Vaccination Coverage in Quebec, Canada, Displayed by Month and Predominant Variant-of-Concern Periods

eFigure 2. Exposure Categories Defined by Prior Primary SARS-CoV-2 Infection and Vaccination Histories

eFigure 3. Participant Flowchart

eFigure 4. Estimated Protection of Primary SARS-CoV-2 (Non-Omicron) Infection Against Omicron Reinfection, by Time Since Last Event (Primary Infection or Vaccination), Relative to No Vaccination and No Infection History

This supplementary material has been provided by the authors to give readers additional information about their work.

© 2022 Carazo S et al. *JAMA Network Open*.

eTable. Estimated Vaccine Protection Against Omicron Reinfection Among Individuals With Prior SARS-CoV-2 Infection Stratified by Age and Primary Infection Characteristics, Relative to Nonvaccinated Individuals With Primary SARS-CoV-2 Infection

	**PI-V1**	**PI-V2**	**PI-V3**
	Adjusted effectiveness[a] (95% CI)	Adjusted effectiveness[a] (95% CI)	Adjusted effectiveness[a] (95% CI)
**Global**	40% (33, 46)	45% (40, 50)	70% (67, 73)
**Age (years)**			
12-17	46% (14, 66)	49% (21, 66)	89% (7, 99)
18-49	39% (31, 46)	46% (39, 52)	69% (64, 73)
50-69	42% (24, 55)	44% (29, 55)	74% (66, 80)
≥70	60% (23, 79)	45% (12, 65)	66% (44, 80)
**VOC status of prior PI**			
non-VOC	45% (38, 52)	53% (47, 59)	75% (72, 78)
Alpha	55% (37, 68)	56% (39, 68)	88% (74, 94)
Delta	22% (-27, 52)	67% (20, 87)	NE
Other / unknown[b]	40% (21, 54)	38% (22, 52)	77% (66, 85)
**Severity of prior PI**			
Asymptomatic	41% (19, 56)	41% (22, 55)	66% (53, 76)
Symptomatic non-hospitalized	39% (32, 46)	46% (40, 51)	71% (67, 74)
Symptomatic hospitalized	36% (-17, 65)	34% (-10, 60)	63% (32, 80)
**Interval since PI**			
3-5 months	30% (-4, 53)	66% (28, 84)	NE
6-8 months	53% (34, 67)	55% (38, 68)	84% (54, 94)
9-11 months	46% (31, 57)	48% (36, 59)	77% (69, 84)
12-18 months	45% (35, 53)	53% (45, 59)	74% (70, 79)
19-24 months	50% (34, 62)	56% (44, 66)	75% (67, 81)
**Severity of and interval since PI**			
Asymptomatic <6 months	62% (-94, 93)	NE	NE
Asymptomatic ≥6 months	47% (26, 62)	48% (31, 62)	71% (59, 79)
Symp non-hosp <6 months	30% (-9, 55)	66% (24, 85)	NE
Symp non-hosp ≥6 months	45% (38, 52)	51% (45, 57)	74% (70, 77)
Symp hosp <6 months	NE	NE	NE
Symp hosp ≥6 months	47% (-3, 73)	47% (6, 70)	70% (42, 85)
**Interval since last vaccination**[c]			
<2 months	67% (55, 76)	72% (67, 76)	73% (69, 76)
2-5 months	40% (32, 47)	44% (38, 49)	65% (57, 72)
6-8 months	37% (28, 44)	38% (31, 45)	NE
9-11 months	39% (25, 50)	38% (2, 60)	NE
12-14 months	54% (31, 70)	NE	NE

[a] Logistic regression models comparing persons with prior primary infection with vaccination to those with prior infection unvaccinated. All estimates adjusted for age (4 categories), sex, indication for testing and epidemiological week.

[b] Cases without genotyping during periods with mixed circulation or cases from beta variant (n=2) or gamma variant (n=5)

[c] Models stratified for delay from last vaccination are not adjusted for epidemiological week due to insufficient number of cases in each strata and high correlation between delay and epidemiological week for those vaccinated with 3 doses.

Abbreviations: CI, confidence interval; hosp, hospitalized; NE, non-estimable; PI, prior primary infection; PI-V1, prior infection before one vaccine dose; PI-V2, prior infection before two vaccine doses; PI-V3, prior infection before three vaccine doses; symp, symptomatic; VOC, variant of concern

© 2022 Carazo S et al. *JAMA Network Open*.

eFigure 1. Epidemiologic Curve of Reported SARS-CoV-2 Cases and Vaccination Coverage in Quebec, Canada, Displayed by Month and Predominant Variant-of-Concern Periods

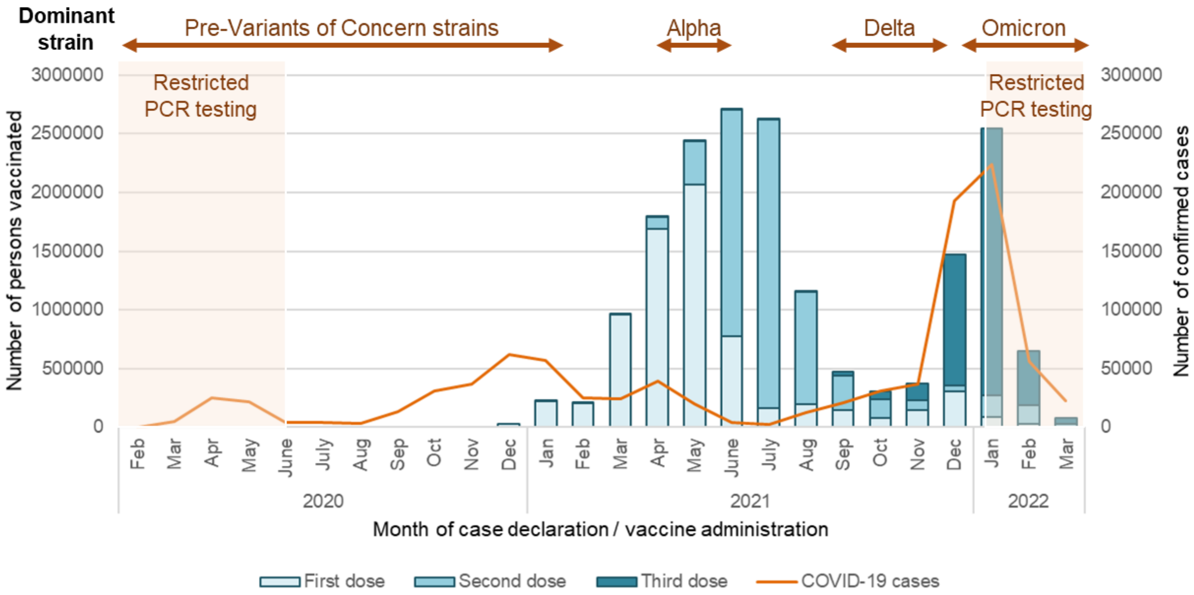

Note: Variant-of-concern (VOC) detection provincially varied over the course of the pandemic in response to changing epidemic patterns, case load and laboratory capacity as well as the profile of emerging and identified VOC. Predominant VOC periods were defined by provincial genomic-based surveillance and were also used to assign likely individual VOC case status.

Before February 2021, VOC detection was undertaken on convenience samples by whole genome sequencing (WGS). From February 1, 2021 to mid-October 2021 screening was systematically undertaken on all specimens. From October 2021 to February 2022 screening was undertaken on a random sample of 5-10% of case viruses, prioritizing travelers, reinfections, outbreaks and severe cases. Between October 9 and December 12, 2021 Delta was almost exclusively identified among characterized viruses. On November 30 and December 14, 2021 all case viruses were screened with Omicron detected in none and 20% of samples, respectively.[1] Conversely, during the first two weeks of the study period after December 25, 2021, 94% and 98% of cases viruses from sentinel laboratories that were genetically screened were Omicron. The BA.2 sub-lineage of Omicron was first detected in Quebec at the end of January 2022, representing 53% of screened specimens by mid-March 2022.[1]

Based on the above, individual VOC status of primary infections occurring before the study period and of cases identified during the study period was assigned as follows:

- Before February 1, 2021: assumed pre-VOC
- February 1 to October 8, 2021: Alpha/Beta/Gamma/Delta as individually diagnosed
- October 9 to December 12, 2021: assumed Delta
- December 13-25, 2021: excluded based on requirement for ≥90-day delay (see study exclusions)
- December 26, 2021 to March 12, 2022 (study period): assumed Omicron

[1] Institut national de santé publique du Québec. Données sur les variants du SRAS-CoV-2 au Québec. Published April 1, 2022. Accessed April 1, 2022. https://www.inspq.qc.ca/covid-19/donnees/variants

© 2022 Carazo S et al. *JAMA Network Open*.

eFigure 2. Exposure Categories Defined by Prior Primary SARS-CoV-2 Infection and Vaccination Histories

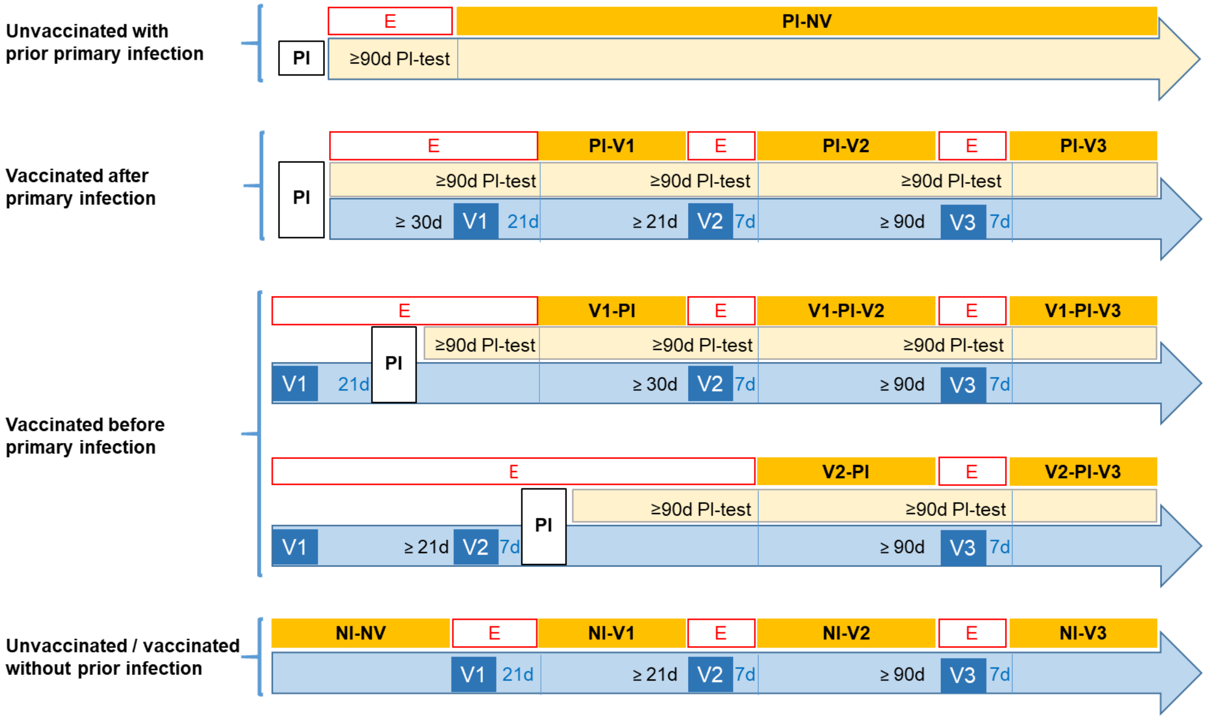

Abbreviations: d, days (between vaccine doses in blue font and between primary infection, testing and vaccine doses in black font); E, exclusion; NI, no infection previously; NI-NV, no prior infection previously non-vaccinated; NI-V1, no infection previously, one vaccine dose; NI-V2, no infection previously, two vaccine doses; NI-V3, no infection previously, three vaccine doses; PI, prior primary infection; PI-NV, prior infection non-vaccinated; PI-V1, prior infection before one vaccine dose; PI-V2, prior infection before two vaccine doses; PI-V3, prior infection before three vaccine doses; V1-PI, prior infection after one vaccine dose; V1-PI-V2, prior infection after first but before second vaccine dose; V1-PI-V3, prior infection after first but before second and third vaccine doses; V2-PI, prior infection after two vaccine doses; V2-PI-V3, prior infection after second but before third vaccine dose; V3-PI, prior infection after three vaccine doses; V1, vaccine dose 1; V2, vaccine dose 2; V3, vaccine dose 3

© 2022 Carazo S et al. *JAMA Network Open*.

eFigure 3. Participant Flowchart

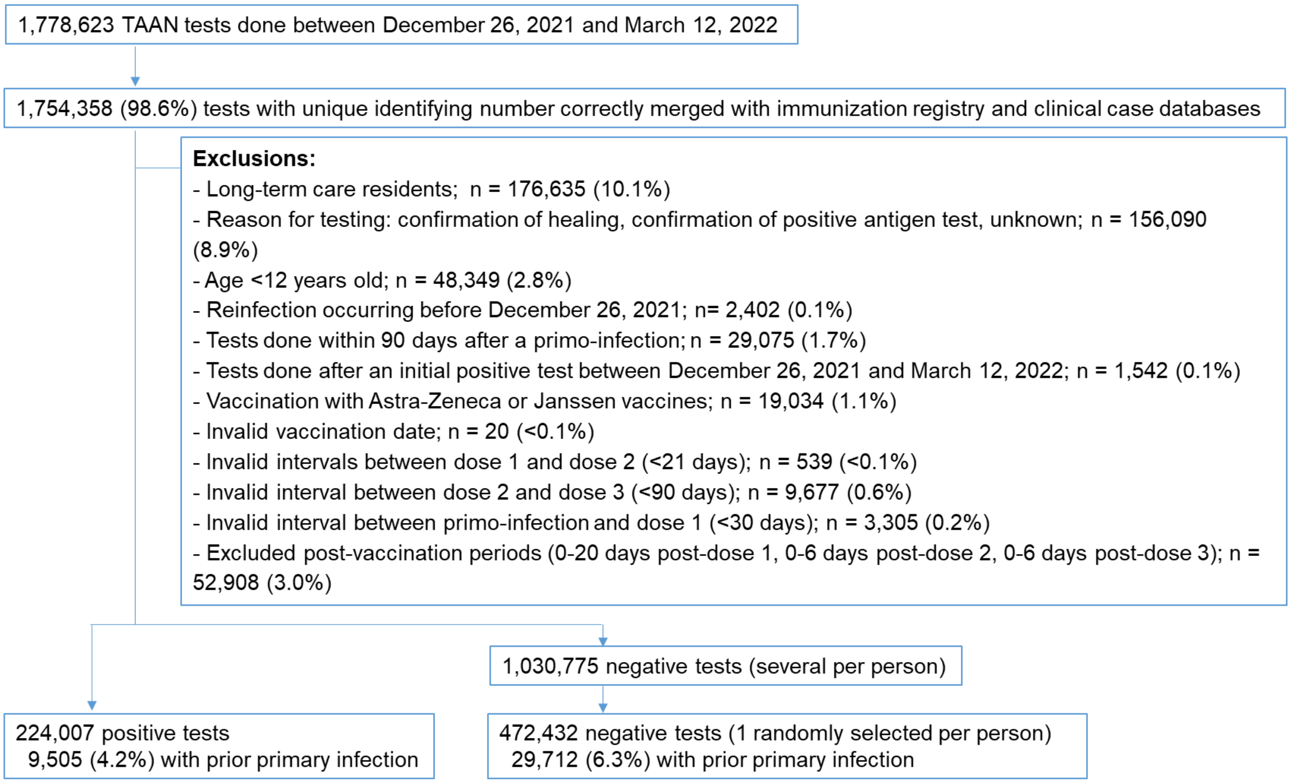

© 2022 Carazo S et al. *JAMA Network Open*.

eFigure 4. Estimated Protection of Primary SARS-CoV-2 (Non-Omicron) Infection Against Omicron Reinfection, by Time Since Last Event (Primary Infection or Vaccination), Relative to No Vaccination and No Infection History

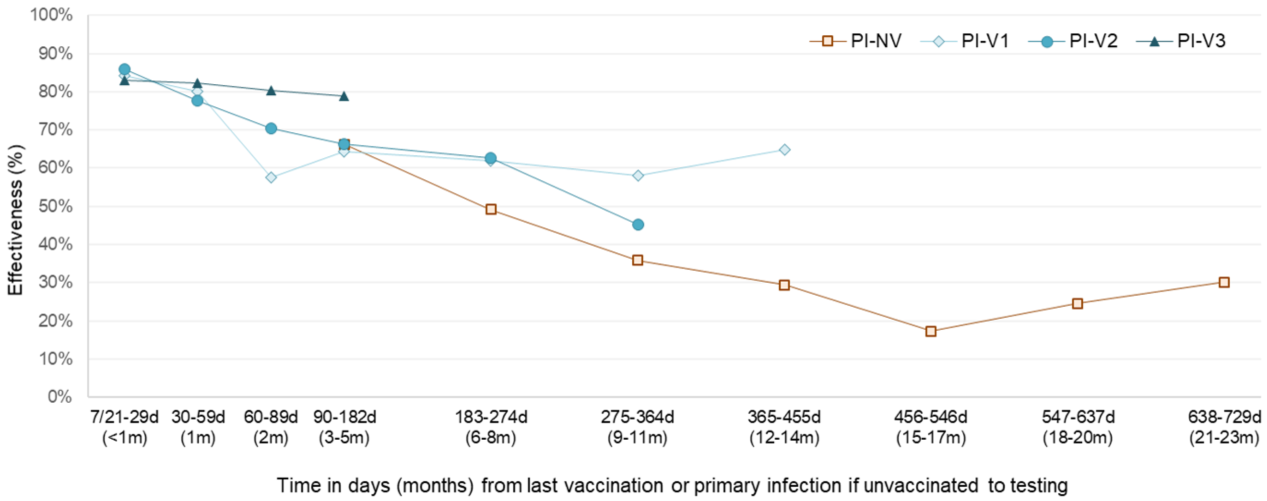

Abbreviations: d, days; m, months; PI-NV, prior infection non-vaccinated; PI-V1, prior infection before one vaccine dose; PI-V2, prior infection before two vaccine doses; PI-V3, prior infection before three vaccine doses

Note: Logistic regression models comparing persons with prior primary infection with/without vaccination to those unvaccinated and without prior infection. All estimates adjusted for age (4 categories), sex, indication for testing and epidemiological week

© 2022 Carazo S et al. *JAMA Network Open*.
